# Characterization of an Italian Founder Mutation in the RING-Finger Domain of *BRCA1*

**DOI:** 10.1371/journal.pone.0086924

**Published:** 2014-02-06

**Authors:** Laura Caleca, Anna Laura Putignano, Mara Colombo, Caterina Congregati, Mohosin Sarkar, Thomas J. Magliery, Carla B. Ripamonti, Claudia Foglia, Bernard Peissel, Daniela Zaffaroni, Siranoush Manoukian, Carlo Tondini, Monica Barile, Valeria Pensotti, Loris Bernard, Laura Papi, Paolo Radice

**Affiliations:** 1 Unit of Molecular Bases of Genetic Risk and Genetic Testing, Department of Preventive and Predictive Medicine, Fondazione IRCCS Istituto Nazionale dei Tumori (INT), c/o Amadeolab, Milano, Italy; 2 Department of Biomedical, Experimental and Clinical Sciences, University of Florence, Florence, Italy; 3 FiorGen Foundation for Pharmacogenomics, Sesto Fiorentino, Italy; 4 Department of Chemistry and Department of Biochemistry, The Ohio State University, Columbus, Ohio, United States of America; 5 Unit of Medical Genetics, Department of Preventive and Predictive Medicine, Fondazione IRCCS Istituto Nazionale dei Tumori, Milan, Italy; 6 Unit of Medical Oncology, Azienda Ospedaliera Papa Giovanni XXIII, Bergamo, Italy; 7 Division of Cancer Prevention and Genetics, Istituto Europeo di Oncologia, Milan, Italy; 8 COGENTECH-Cancer Genetic Testing Laboratory, Milan, Italy; 9 Fondazione Istituto FIRC di Oncologia Molecolare, Milan, Italy; 10 Department of Experimental Oncology, Istituto Europeo di Oncologia, Milan, Italy; Odense University Hospital, Denmark

## Abstract

The identification of founder mutations in cancer predisposing genes is important to improve risk assessment in geographically defined populations, since it may provide specific targets resulting in cost-effective genetic testing. Here, we report the characterization of the *BRCA1* c.190T>C (p.Cys64Arg) mutation, mapped to the RING-finger domain coding region, that we detected in 43 hereditary breast/ovarian cancer (HBOC) families, for the large part originating from the province of Bergamo (Northern Italy). Haplotype analysis was performed in 21 families, and led to the identification of a shared haplotype extending over three *BRCA1*-associated marker loci (0.4 cM). Using the DMLE+2.2 software program and regional population demographic data, we were able to estimate the age of the mutation to vary between 3,100 and 3,350 years old. Functional characterization of the mutation was carried out at both transcript and protein level. Reverse transcriptase-PCR analysis on lymphoblastoid cells revealed expression of full length mRNA from the mutant allele. A green fluorescent protein (GFP)-fragment reassembly assay showed that the p.Cys64Arg substitution prevents the binding of the BRCA1 protein to the interacting protein BARD1, in a similar way as proven deleterious mutations in the RING-domain. Overall, 55 of 83 (66%) female mutation carriers had a diagnosis of breast and/or ovarian cancer. Our observations indicate that the *BRCA1* c.190T>C is a pathogenic founder mutation present in the Italian population. Further analyses will evaluate whether screening for this mutation can be suggested as an effective strategy for the rapid identification of at-risk individuals in the Bergamo area.

## Introduction

Inactivating germline mutations of *BRCA1* and *BRCA2* genes account for approximately 15% to 20% of hereditary breast and/or ovarian cancer (HBOC) cases [1], [2]. The majority of alterations identified throughout the whole sequence of both genes are private or detected in few families only [3], but a number of specific mutations that appear repeatedly in ethnically defined groups or in populations enriched with genetic isolates, because of a shared common ancestry (founder mutations), have been reported [4].

The identification of founder mutations in various ethnic groups and their geographical distribution has important implications for designing mutational screening. For example, three founder mutations in the Ashkenazi Jewish (*BRCA1* c.68_69delAG, *BRCA1* c.5266dupC, *BRCA2* c.5946delT) and one in the Icelanders (*BRCA2* c.771_775del5), account for virtually all HBOC families linked to BRCA genes in these populations [5]–[8], where, therefore, it is worthwhile to test high-risk patients specifically for these mutations, before considering the more expensive complete sequence analysis of both genes. Furthermore, the identification of recurrent genomic rearrangements of the *BRCA1* and *BRCA2* genes, due to founder effects, has provided a strong rational for including the screening for these alterations in the diagnostic setting. For instance, in the Dutch population two distinct deletions involving exons 13 and 22 account for approximately 25% of all *BRCA1*-positive families [9], while approximately 9% of *BRCA1*-positive families in the United Kingdom carry a common exon 13 duplication [10]. As a result, in these populations screening for specific *BRCA1* rearrangements is now part of routine genetic testing of HBOC families. Notably, the knowledge of founder *BRCA1* or *BRCA2* mutations may provide more precise estimates of the prior probability of carrying a mutation in either genes and of the likelihood of a mutation carrier developing cancer [11].

To date, a few BRCA founder mutations have been reported also in the Italian population, where they appear to be limited to geographically restricted areas (reviewed in ref. 4).

Here, we report the characterization of the *BRCA1* c.190T>C missense mutation (p.Cys64Arg), located in the gene region coding for the RING-finger motif of the protein, which segregates with the disease in Italian HBOC families, mostly originating from the province of Bergamo (Northern Italy).

## Materials and Methods

### Ethics Statement

All subjects included in the study received genetic counseling and provided a written informed consent for BRCA gene mutation testing and for the use of their biological samples for research purposes: This study was approved by the ethical committee of the Fondazione IRCCS Istituto Nazionale dei Tumori (INT) of Milan.

### Case Material

The study includes 43 apparently unrelated families carrying the c.190T>C (p.Cys64Arg) mutation in *BRCA1* exon 5, identified among those referred for BRCA gene testing from December 1995 to December 2012 by the following institutions: Fondazione IRCCS Istituto Nazionale dei Tumori (INT) and Istituto Oncologico Europeo (IEO) of Milan, Azienda Ospedaliera Papa Giovanni XXIII of Bergamo (AO-BG) and University of Florence (Department of Biomedical, Experimental and Clinical Sciences). The presence of the mutation was ascertained in family probands by either denaturing high performance liquid chromatography (DHPLC) and/or direct sequencing of all coding exons and adjacent intronic regions of the *BRCA1* and *BRCA2* genes. Sequencing of *BRCA1* exon 5 was performed to identify additional mutation carriers among the probands' relatives.

### Microsatellite analysis

A total of 76 individuals, including 43 mutation carriers and 33 non carriers, from 21 of the above families were genotyped at the *BRCA1* locus as previously described [12].

### Haplotyping and estimate of mutation age

Haplotypes were constructed manually from microsatellite analyses, assuming the least number of possible recombinations. Age estimate of the *BRCA1* c.190T>C mutation was carried out with the DMLE+2.2 software program (URL: http://www.dmle.org) [13]. The DMLE input file included the full haplotypes of mutations carriers and of non-carriers, used as controls from the general population, for the 6 examined markers, chromosome map distances derived from the Marshfield and/or Genéthon sex-average genetic maps (URL: http://www.ncbi.nlm.nih.gov/mapview/), an estimate of population growth rate per generation and an estimate of the proportion of sampled mutation-carrying chromosomes. The population growth rates per generation (*r*) were estimated as described [12], based on available demographic data (Italian National Statistics Institute, ISTAT; URL: http://www.istat.it) and assuming a time interval of 25 years per generation. The *r* value for the province of Bergamo from the year 1300 to present was estimated to be 0,0526. This index was subsequently used for mutation age estimates. Three separate analyses were then performed, each using a different estimate for the proportion of sampled mutation-carrying chromosomes: 0.015, 0.01, and 0.005.

### Transcript analysis

Epstein-Barr virus (EBV)-immortalized human lymphoblastoid cell lines (LCLs) were established and cultured as described [14]. Four *BRCA1* mutant and six wild-type LCLs were grown in the absence and in the presence of cycloheximide (CHX) (100 µg/ml) for 4 hours to account for potential degradation of unstable transcripts via nonsense mediated mRNA decay. Total RNA was purified and reverse transcribed into cDNA as described [14]. The PCR reaction was performed with a forward primer in *BRCA1* exon 5 (5′-GCATGCTGAAACTTCTCAAC-3′), and a reverse primer in exon 6 (5′-TCCAAACCTGTGTCAAGCTG-3′). The reverse primer was labeled with 6-carboxyfluorescein (6-FAM). The fluorescent amplification products were run on a 3130 Genetic Analyzer (Applied Biosystems) using the GeneScan 500 ROX Size Standard (Applied Biosystems) as internal marker. Size calling and quantification of peak areas were performed with GeneMapper Software v4.0 (Applied Biosystems). The molecular nature of the peaks was confirmed by sequencing.

### Statistical Analysis

The ratios of the peak areas of the *BRCA1* Δexon5q and full-length mRNA isoforms in different samples were compared by two-tailed Student t test using GraphPad Prism version 5.0 software and 95% confidence interval (CI).

### Plasmid construction

The pET11a-NfrGFP-Z and pMRBAD-Z-CfrGFP expression vectors, encoding anti-parallel leucine zipper motifs (Z) fused to the N-terminal or C-terminal fragment of Green Fluorescent Protein (NfrGFP, CfrGFP, respectively) and the plasmids encoding N-terminal RING domains of BARD1 (amino acids 26–140) and BRCA1 (amino acids 1–109) attached via a linker sequences to the NfrGFP (pET11a-NfrGFP-BARD1) or CfrGFP (pMRBAD-BRCA1-CfrGFP), were created as described [15]. The pET11a-NfrGFP-Z and pET11a-NfrGFP-BARD1 vectors also encode a hexahistidine (H_6_)-tag at the N-terminus of the NfrGFP useful for rapid purification of the H_6_-tagged protein. The pMRBAD-Z-CfrGFP and pMRBAD-BRCA1-CfrGFP (both as wild-type and mutant forms) bear an HA epitope at the C-terminus of the CfrGFP as a detection tag. The *BRCA1* c.190T>C (p.Cys64Arg), c.199G>T (p.Asp67Tyr) and c.181T>G (p.Cys61Gly) mutants were obtained by direct mutagenesis of pMRBAD-BRCA1-CfrGFP using the QuikChange XL Site-directed Mutagenesis Kit (Stratagene) according to the manufacturer's instruction. Recombinant clones were checked by DNA sequencing.

### GFP-fragment reassembly screening

Compatible pairs of plasmids [pET11a-NfrGFP-Z and pMRBAD-Z-CfrGFP; pET11a-NfrGFP-BARD1 and pMRBAD-BRCA1-CfrGFP (both as wild-type or mutant forms)] were co-transformed into BL21-(DE3) *E. coli* competent cells by electroporation. Single colonies were screened for the occurrence of GFP-fragment reassembly as previously described [15].Fluorescence was observed after excitation with long-wave (365 nm) UV light in combination with the short pass (SP) emission filter using a Syngene image capture system (SYNGENE) as specified by the manufacturer.

### Purification of the reassembled GFP complexes

The H_6_-NfrGFP-Z/Z-CfrGFP-HA and H_6_-NfrGFP-BARD1/BRCA1-CfrGFP-HA (both as wild-type or mutant forms) complexes were purified from the soluble fraction of co-transformed *E. coli* strain BL21-(DE3) by Immobilized Metal Affinity Chromatography (IMAC) using nickel nitrilotriacetic (Ni-NTA) agarose resin (QIAGEN), following the protocol described [15]. The protein complexes were subjected to 13% SDS-PAGE and visualized by Western blotting using a polyclonal anti-GFP antibody (# 600-101-215; Rockland). Unpurified cell lysates from induced *E. coli* BL21-(DE3) bacteria were similarly resolved and visualized to detect expression levels of both NfrGFP-BARD1 and CfrGFP-BRCA1 recombinant proteins.

### Clinical and pathological characteristics

Clinical and pathological data of affected mutation carriers were collected at genetic counseling and from medical records. Loss of heterozygosity (LOH) at *BRCA1* locus in tumor DNA was performed as previously described [16].

## Results

### Geographical distribution and frequency of the *BRCA1* c.190T>C families

**Figure 1:**
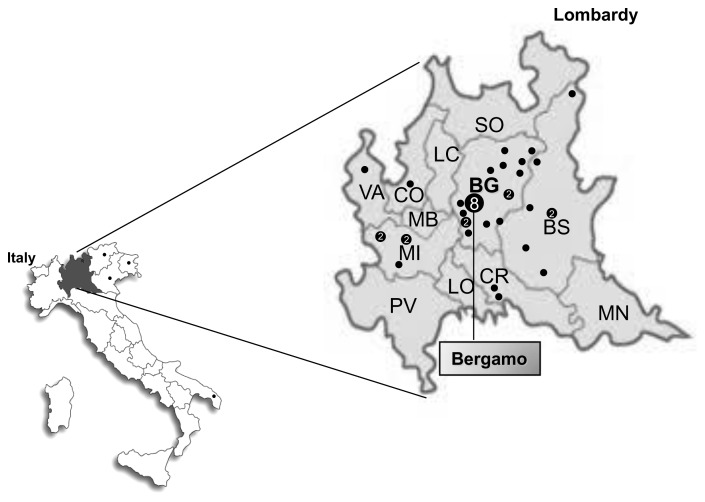
Geographical distribution of *BRCA1* c.190T>C (p.Cys64Arg) mutation carriers. Symbol (“•”) indicates the birth places of index case from families segregating the mutation.

The birth places of the probands of the 43 *BRCA1* c.190T>C positive families are shown in Figure 1. The majority were from the city of Bergamo and its province (n = 23) or from neighboring provinces of the Lombardy region (n = 16). The frequencies of c.190T>C positive families on the total number of families tested for *BRCA1* and *BRCA2* mutations and on the total number of BRCA-positive families were 8.7% and 30.2%, respectively, in cases recruited in Bergamo and 0.8% and 3.2%, respectively, in cases recruited in Milan (Table 1).

**Table 1:** Number and frequencies of *BRCA1* c.190T>C positive families among those recruited at three Italian institutions and tested for BRCA mutations.

Institution	No. identified	% on total tested families*	% on BRCA1/2 families
**INT**	22	1,03 (22/2140)	3,98 (22/553)
**IEO**	4	0,39 (4/1013)	1,54 (4/260)
**INT+IEO**	26	0,82 (26/3153)	3,20 (26/813)
**AO-BG**	16	8,74 (16/183)	30,19 (16/53)

* Intake criteria for BRCA testing are described in Manoukian et al [38].

### Haplotype analysis

**Figure 2:**
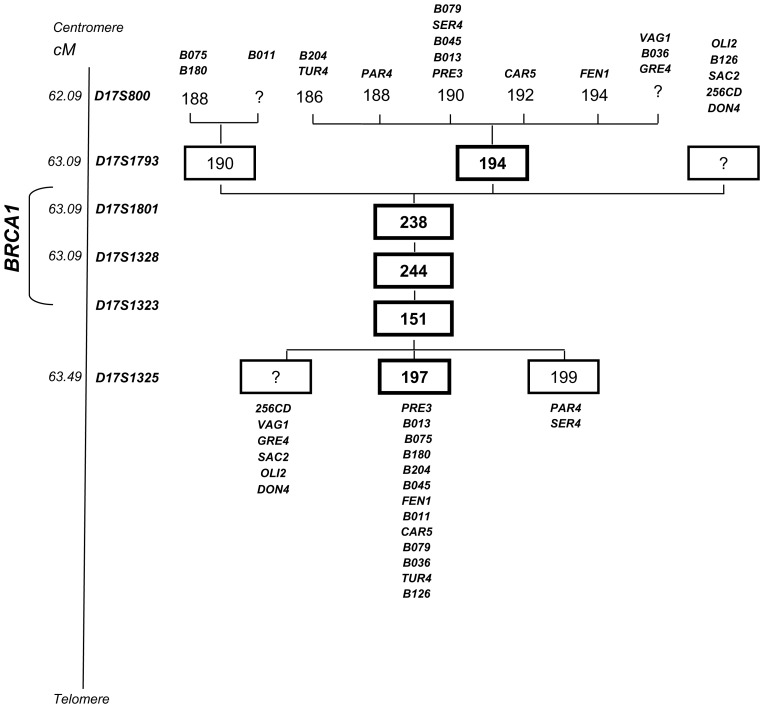
Haplotype branching trees in families segregating the *BRCA1* c.190T>C (p.Cys64Arg). The six short tandem repeat markers analyzed are shown together with their position in the Marshfield genetic map. Family haplotypes are indicated with the corresponding family ID codes. The most common haplotype is indicated in bold numbers.

Fragment analysis of 6 microsatellite marker loci intragenic and flanking *BRCA1* identified a shared haplotype extending over 3 marker loci (0.4 cM) in carriers of the *BRCA1* c.190T>C from 20 families for which DNA of more than one member was available (Figure 2). In one additional mutated family with only one individual available for the analysis, the observed genotypes were compatible with the shared haplotype.

### Age estimate of the *BRCA1* c.190T>C mutation

The DMLE+2.2 program was used to estimate the age of the *BRCA1* c.190T>C mutation based on haplotype data from mutation carriers and non carriers.

Three separate analyses were performed, using a population growth rate per generation of 0.052 and different estimates of the proportion of sampled mutation-carrying chromosomes (0.015, 0.01 and 0.005). The resulting age estimates were 124 generations (95% credible set: 79–170), 130 generations (95% credible set: 91–170) and 134 generations (95% credible set: 97–171), respectively (data not shown). Assuming an interval of 25 years per generation, this corresponds to the mutation being 3,100, 3,250 and 3,350 years old, respectively. Thus, age estimates were only slightly affected by the actual proportion of sampled mutation-carrying chromosomes.

### Evaluation of the effect of the *BRCA1* c.190T>C variant on mRNA transcripts

**Figure 3:**
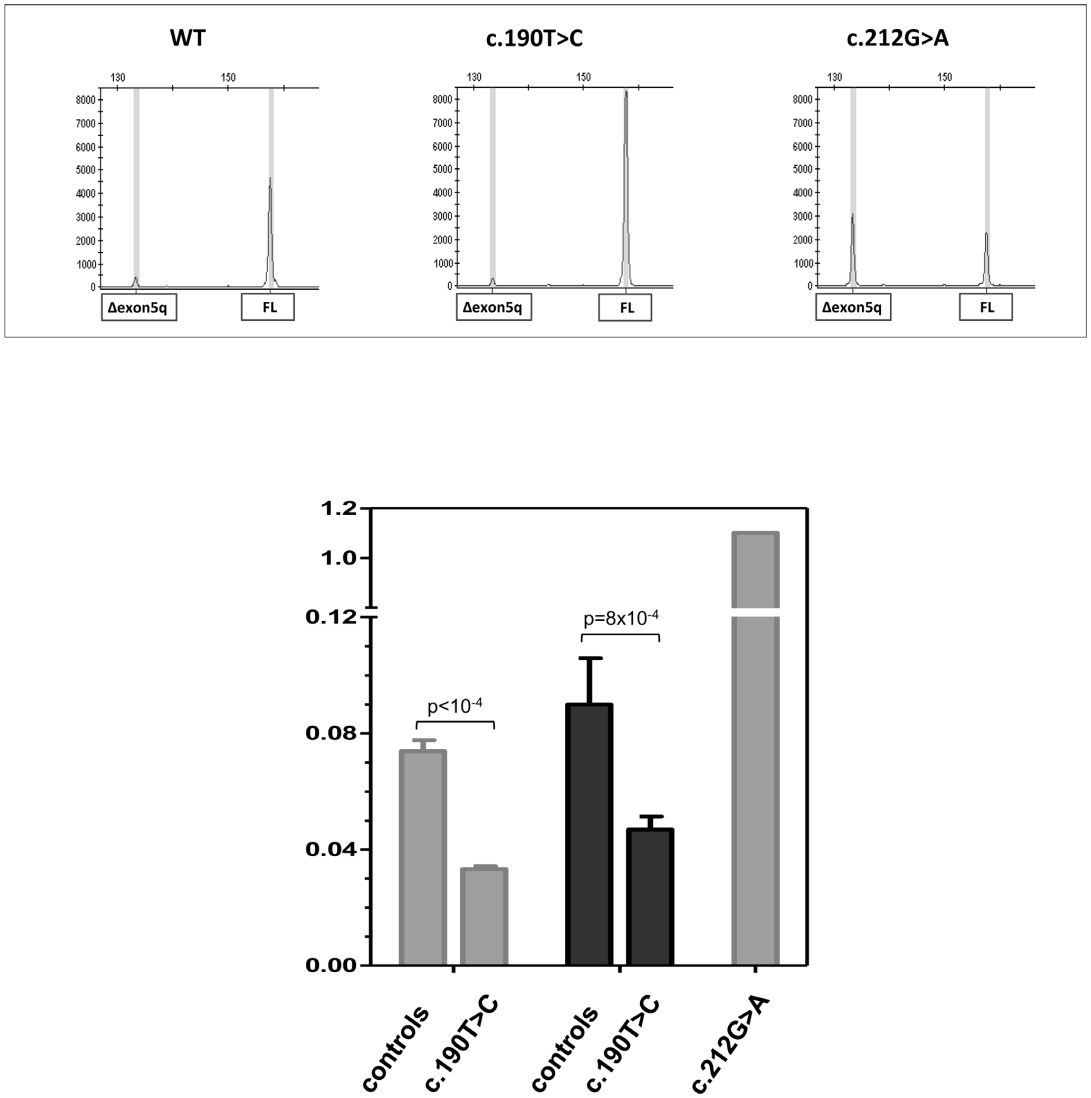
Semi-quantitative fragment analysis of the Δexon5q isoform. The upper panel shows the capillary electrophoresis patterns of the cDNA fragments spanning *BRCA1* exons 5 and 6 observed in LCLs from a *BRCA1* wild type individual, and from carriers of the c.190T>C and c.212G>A, which causes the up-regulation of the Δexon5q transcript, mutations. The Δexon5q and full-length (FL) isoforms are indicated. The lower panel shows the ratio between the peak areas of the Δexon5q and full-length isoforms. The LCLs were cultured in the presence (dark grey bars) and in the absence (light grey bar) of cycloheximide. Control bars represent the average value observed in six wild-type LCLs. c.190T>C bars represent the average value observed in four mutant LCLs. The error bars represent standard deviation.

The *BRCA1* c.190T>C variant is mapped to an alternatively used donor splice site. The usage of this site leads to the synthesis of a naturally occurring isoform missing 22 bp at the 3′-end of exon 5 (Δexon5q) [17], [18]. To evaluate the putative effect of the mutation on Δexon5q and full-length transcription levels, the cDNA region encompassing the mutation site was amplified and analyzed by capillary electrophoresis. The ratio between the peak areas of the Δexon5q and the full-length isoforms in LCLs carrying the c.190T>C, cultured in the presence and in the absence of cycloheximide, was calculated and compared with those of wild-type LCLs (Figure 3). The LCL carrying the *BRCA1* c.212G>A mutation previously reported to up-regulate the Δexon5q isoform [14] was used as internal control. A significant decrease in the ratio of the Δexon5q vs. full length expression levels was observed in the c.190T>C LCLs compared to normal controls (0,45, 95% CI = 0.44–0.46 and 0.52, 95%, CI = 0.50–0.54, for the CHX-untreated and treated samples, respectively). Sequence analyses detected the presence of the mutation in the full length cDNA (data not shown). These results indicate that the c.190T>C mutation does not impair the synthesis of full-length mRNA and are consistent with a sustained expression of the mutated protein.

### Evaluation of the effect of the c.190T>C mutation on the interaction of BRCA1 with BARD1

The BRCA1 protein displays an E3 ubiquitin ligase activity mediated by the interaction with BARD1 through its N-terminal RING-finger domain [19], [20], where the c.190T>C (p.Cys64Arg) is located.

To assess whether the mutation affects the BRCA1/BARD1 complex formation, we carried out a GFP-fragment reassembly screening, a Bimolecular Fluorescence Complementation-based assay (BiFC) [21]. In this assay, the GFP is dissected into two fragments (NfrGFP and CfrGFP) that, when expressed together in *E. coli* cells, do not spontaneously reassemble into a fluorescence protein. However, if the two fragments of GFP are each individually fused to two interacting proteins, this interaction can mediate reassembly of the GFP in co-transformed bacteria with consequent cellular fluorescence [22].

**Figure 4:**
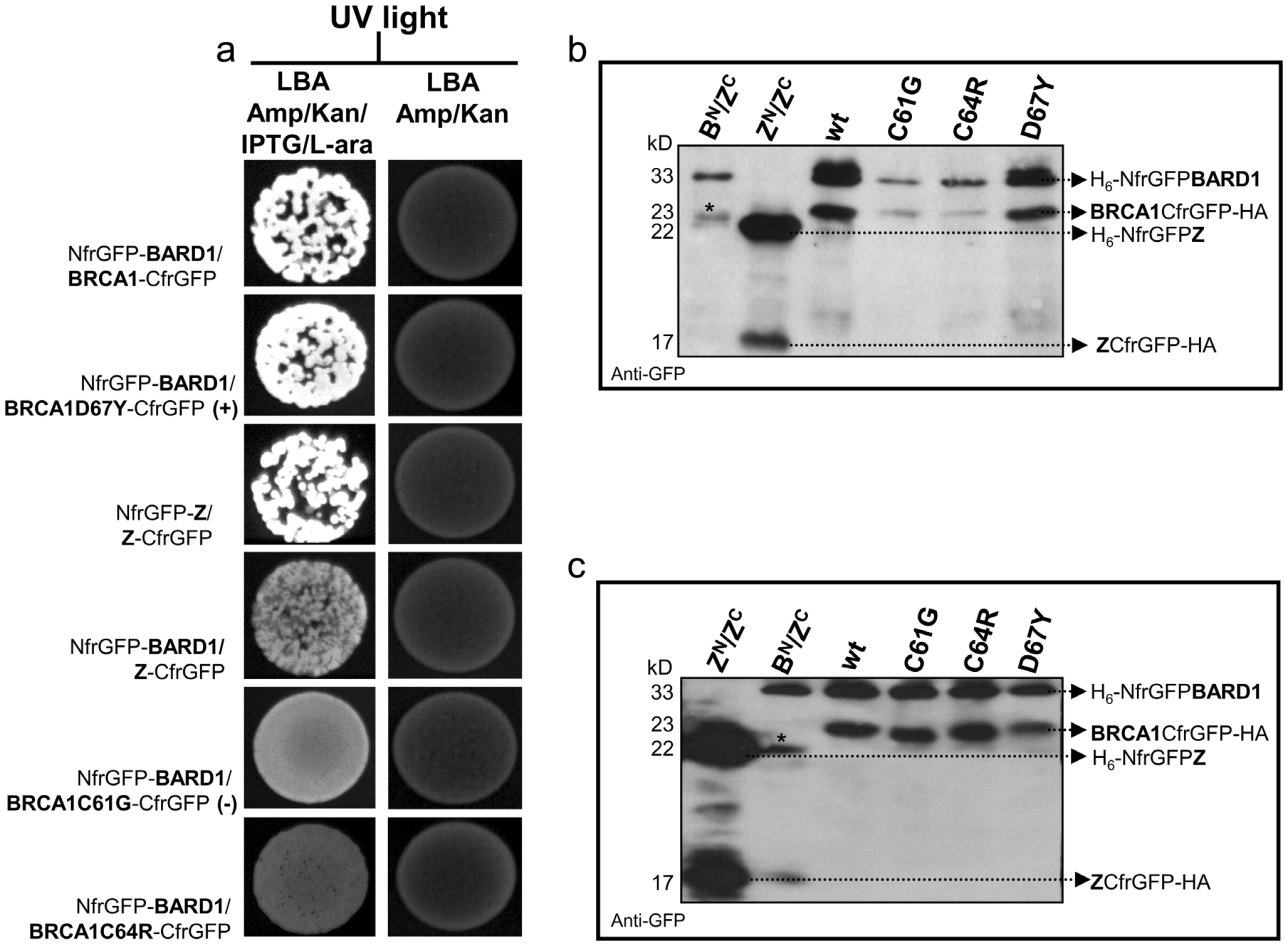
Detection of BRCA1/BARD1 interaction by GFP-fragment reassembly screening. (a) Fluorescence was observed after 24 h of growth at 30°C followed by 2 days of incubation at RT. No fluorescence is observed under non-inducing condition (right column). [L-ara, L-arabinose; IPTG, Isopropyl β-D-1-tiogalattopiranoside, IPTG]. (b) SDS-PAGE of purified, reassembled complexes by IMAC methods. The expected molecular masses are indicated on the left. [*Non-specific band. B^N^, H_6_-NfrGFPBARD1; Z^N^, H_6_-ZNfrGFP; Z^C^, ZCfrGFP-HA]. (c) Expression of NfrGFP-BARD1 and CfrGFP-BRCA1 wild-type and mutant forms.

Under inducing conditions (0.2% L-arabinose and 20 µM IPTG; Fig. 4A, left column), bright fluorescence was observed in bacterial cells co-expressing BARD1-NfrGFP together with BRCA1-wild type/CfrGFP or BRCA1-D67Y/CfrGFP carrying the variant p.Asp67Tyr, previously classified as clinically neutral [3] and the strong interacting anti-parallel Z-NfrGFP/Z-CfrGFP fusion peptides. No fluorescence was observed in bacteria co-expressing the following fusion peptides: non cognate BARD1 NfrGFP/CfrGFP-Z, BARD1-NfrGFP/BRCA1-C61G-CfrGFP carrying the p.Cys61Gly disease-causing mutation [23], and BARD1-NfrGFP/BRCA1-C64R-CfrGFP carrying the p.Cys64Arg mutation. In addition, IMAC purified reassembled complexes from the soluble fraction of co-transformed cells *E. coli* BL21-(DE3) were analyzed by Western blotting (Figure 4B). Using a polyclonal anti-GFP antibody, two strong bands corresponding to the components of the GFP reassembled complexes were detected in lysates of bacterial cells co-expressing Z-NfrGFP/CfrGFP-Z and BARD1-NfrGFP together with BRCA1-wild type/CfrGFP or BRCA1-Asp67Tyr. Much more reduced bands were observed in cell lysates co-expressing BARD1-NfrGFP and BRCA1-Cys61Gly/CfrGFP or BRCA1-Cys64Arg/CfrGFP. Since unassembled GFP fusion fragments are unfolded and less soluble [24], these results indicate GFP reassembly and, therefore, BRCA1/BARD1 binding for the BRCA1-wt and BRCA1-Asp67Tyr constructs, but not for the BRCA1-Cys61Gly and BRCA1-Cys64Arg constructs. These observations are consistent with those obtained by fluorescence complementation assay. Unpurified cell lysates from co-transformed *E. coli* BL21-(DE3) bacteria, were visualized by Western blotting with a polyclonal anti-GFP antibody, revealing that all the fusion peptides were expressed to a similar extent (Figure 4C). This demonstrates that the lack of co-purification of CfrGFP-BRCA1-HA fragments for the Cys61Gly and Cys64Arg constructs is attributable to the lack of binding to BARD1 and not to poor expression of the BRCA1 mutants.

### Clinical and pathological features of *BRCA1* c.190T>C carriers

Overall, a total of 86 ascertained mutation carriers and of 7 obligate carriers were identified in the families recruited in the study, including 83 females and 10 males. Among female carriers, 55 (66%) reported a diagnosis of breast carcinoma (n = 38), ovarian carcinoma (n = 12) or both breast and ovarian carcinomas (n = 5).

Available clinical and pathological features of the affected female carriers are reported in Table 2 and 3. The median ages of breast cancer and ovarian cancer diagnosis were 39.6 and 48.2 years, respectively. The large majority of breast cancers were of high grade (23/28 = 82.1%). The prevalent histological type was ductal (32/38 = 84.2%), being the remaining cases mostly of the medullary type (4/38 = 10.5%). The frequencies of cancers positive for estrogen receptor (ER), progesterone receptor (PR) and human epidermal growth factor receptor 2 (HER2/Neu) were 4/31 (12.9%), 6/31 (19.4%) and 3/22 (13.6%), respectively. The information for all the above tumor markers was available in 23 cases, and the majority (15 = 65.2%) displayed a triple-negative (TN) phenotype (lack of the expression of ER, PR and HER2/Neu). The frequencies of contralateral breast cancer were 8/18 (44.4%) and 12/15 (80%) 5 and 10 years after the first diagnosis of breast cancer, respectively. Three patients were diagnosed with additional cancers, including one malignant mixed mullerian tumor (MMMT) of the endometrium, one endometrial carcinoma, and one basal cell carcinoma. As for ovarian cancers, the large majority were of high grade (10/11 = 90.9%) and of the serous type (9/11 = 81.8%). Of the remaining female carriers, one reported a pheochromocytoma at 27 years, and 27 were cancer-free at the time of last contact (mean age = 41 years).

**Table 2:** Clinical and pathological features of *BRCA1* c.190T>C related breast cancer cases.

Breast Cancers (n = 43)		
	n	%
***Age at first diagnosis (years)***		
<36	15	34.9
36	22	51.2
>50	6	14.0
Median	39.6	
***Behaviour***		
Invasive	40	100
In situ	-	-
Not available	3	
***Histological types***		
Ductal	32	84.2
Lobular	-	-
Medullary	4	10.5
Combined^a^	1	2.6
Other	1	2.6
Not available	5	
***Histological grade***		
1	2	7.1
2	3	10.7
3	23	82.1
Not available	15	
***ER***		
Positive	4	12.9
Negative	27	87.1
Not available	12	
***PR***		
Positive	6	19.4
Negative	25	80.6
Not available	12	
***Her2/Neu***		
Positive	3	13.6
Negative	19	86.4
Not available	21	
***Triple Negative***		
Yes	15	65.2
Not	8	34.8
Not available	20	

a Ductal and lobular type.

**Table 3:** Clinical and pathological features of *BRCA1* c.190T>C related ovarian cancer cases.

Ovarian cancers (n = 17)		
	n	%
***Age at first diagnosis (years)***		
<36	-	-
36	12	70.6
>50	5	29.4
Median	48.2	
***Behaviour***		
Invasive	13	100
In situ	-	-
Not available	4	
***Histological types***		
Serous	9	81.8
Mucinous	-	-
Endometroid	2	18.2
Clear Cell	-	-
Other	-	-
Not available	6	
***Histological grade***		
1	-	-
2	1	9.1
3	10	90.9
Not available	6	

Three cancer diagnoses were reported in male carriers, one prostate carcinoma, one tumor of the central nervous system and one leukemia.

LOH at *BRCA1* locus was investigated in two tumors, one breast carcinoma and the MMMT. In both cases, the loss of the constitutional wild-type allele was observed (data not shown).

## Discussion

The *BRCA1* c.190T>C mutation was firstly described in a large Polish cancer family [25]. Subsequently, it was reported in an Italian family characterized by high prevalence of ovarian and breast cancer cases by Willems and co-authors [26]. These authors showed by molecular modeling that the mutation may induce profound modifications in the structure of the BRCA1 RING finger motif, without affecting the normal splicing pattern of the transcripts.

A total of 43 apparently unrelated Italian families carrying the same mutations were referred by four different Italian clinical and university centers. The observation that a large part (23/43 = 53%) were from the same geographic area, the province of Bergamo in Northern Italy, strongly suggested an origin of the mutation from a common ancestor. Consistently, the frequencies of c.190T>C families on the total number of those that underwent BRCA gene testing and of those carrying pathogenic *BRCA1* and *BRCA2* mutations were approximately 10-fold higher in cases recruited in Bergamo, around 85% of whom referred to be born in the local area, compared to those observed in families recruited in two large cancer centers in Milan, which attract patients from all over Italy. The analysis of microsatellite marker loci revealed a shared haplotype in 21 typed families, confirming the founder effect hypothesis. In addition, we estimated the origin of this mutation within a time interval ranging from 3100 to 3350 years ago.

Interestingly, the c.190T>C affects the same nucleotide of another mutation (c.190T>G) that strengthen an alternatively used donor splice site in exon 5 and also disrupts a putative exonic splicing enhancer motif. This leads the loss of the natural donor splice site, resulting in the lack of full length expression and the up-regulation of the naturally occurring Δexon5q isoform, predicted to lead to the synthesis of a truncated protein [17], [18]. In agreement with previous observations [26], no such effect was observed for the c.190T>C. Conversely, in the LCL carrying this mutation a reduction in the relative amount of Δexon5q compared to full length was observed. This finding and the detection of the c.190T>C in the full-length cDNA is consistent with a stable expression of the Cys64Arg protein in mutation carriers.

The BRCA1 p.Cys64Arg mutation is located to the gene region coding for the RING-finger motif of the protein. The RING-finger motif is defined by a conserved pattern of seven cysteine and one histidine residues arranged in an interleaved fashion forming two distinct Zn^2+^-binding sites termed Site I and Site II [27]. Approximately 10% of the clinically relevant mutations of BRCA1 currently reported in the Breast Cancer Information Core (BIC) database (URL: http://research.nhgri.nih.gov/bic/) map within the N-terminal 100 residues-RING domain, which contain the RING motif (residues 23–76). Functional analyses have shown that these mutations abrogate the ubiquitin-ligase activity of BRCA1 by interfering with both the heterodimerization between BRCA1 and BARD1 and the binding of E2 protein UbcH5c to the BRCA1/BARD1 complex [28], [29].

Presently, the BRCA1 p.Cys64Arg mutation is reported in the BIC database as a variant of unknown clinical relevance. However, *in silico* analysis using SIFT (URL: http://sift.jcvi.org/), Polyphen-2 (URL: http://genetics.bwh.harvard.edu/pph2/) and Align-GVGD (URL: http://agvgd.iarc.fr/) bioinformatics tools unanimously predicted the BRCA1 p.Cys64Arg to be deleterious. This is consistent with the notion that Cys64Arg disrupts a critical cysteine residue required for the ubiquitin-ligase activity of BRCA1, through its binding to BARD1 [30]. In fact, the analysis of the three-dimensional Nuclear Magnetic Resonance (NMR) structure of the BRCA1/BARD1 heterodimer showed that p.Cys64Arg substitution determines a profound rearrangement of the BRCA1 39–41 amino acid residues, predicted to result into the impairment of the BRCA1/BARD1 interaction [26].

The result of our GFP-fragment reassembly assay provided experimental evidence that the p.Cys64Arg actually abrogates BRCA1/BARD1 binding as the proven disease causing mutation p.Cys61Gly [31], thus further supporting the pathogenic role of this variant. Interestingly, another mutation affecting the same amino acid residue (p.Cys64Gly) was previously reported to have a similar effect [32]. In addition, our study indicates that this functional approach might be applied to the assessment of the clinical significance of a number of variants in cancer-predisposing genes, implementing currently used strategies.

The median age of breast cancer diagnosis in carriers of the c.190T>C mutation (39.7 years) was similar to that observed in 425 breast cancer patients with other pathogenic *BRCA1* mutations ascertained at the institutions participating in this study (40.1 years). Conversely, among c.190T>C carriers the median age of ovarian cancer onset (48.2 years) was lower than that observed in 192 *BRCA1*-positive ovarian cancer patients from the same institutions (51.9 years), suggesting a possible higher risk of ovarian cancer in carriers of the c.190T>C compared to carriers of other *BRCA1* mutations.

Breast cancers arising in *BRCA1* c.190T>C mutation carriers showed the peculiar histopathological features of the *BRCA1*-related breast tumor [33], [34], with a propensity to be high grade invasive ductal or medullary carcinomas. In addition, the majority displayed a TN phenotype. The clinical relevance of the TN breast cancers is highlighted by the distinctive poor prognosis and no clear options for receptor targeted treatment [35], [36]. In agreement with other reports on *BRCA1* associated ovarian carcinomas [37], [38], invasive epithelial cancer of serous histology was found to be the most common histological subtype among *BRCA1* c.190T>C mutation carriers. Although these observations might be biased by the relative small number of ascertained individuals and by the preferential selection of high risk subjects referred to genetic testing, they are consistent (together with the findings that in two examined tumors the wild type *BRCA1* allele was lost) with the c.190T>C behaving as other *BRCA1* pathogenic variants.

In conclusion, our data show that the *BRCA1* c.190 T>C missense mutation (p.Cys64Arg) is a founder mutation of clinical relevance recurrent in high-risk families from the province of Bergamo, accounting for a significant fraction (ca. 9%) of those ascertained at the local city hospital. Additional studies are needed to assess the actual proportion of carriers of this mutation in this district and its frequency in unselected breast/ovarian cancer patients. This will allow evaluating whether screening for the c.190T>C mutation can be suggested as a cost-effective strategy for the rapid identification of at-risk individuals in the Bergamo area. In addition, they will provide a measure of associated cancer risks, thus improving decision-making regarding clinical management of mutation carriers.
